# Debate on: Laparoscopic cholecystectomy performed by a surgical care practitioner: a review of outcomes by Odogwu *et al*.

**DOI:** 10.1308/rcsann.2024.0103

**Published:** 2024-11-01

In April 2024, following the routine peer review process, the *Annals* published the study entitled *Laparoscopic cholecystectomy performed by a surgical care practitioner: a review of outcomes* by Odogwu *et al.*^1^ This paper subsequently triggered some debate and to date has been downloaded over 15,000 times. As for all published articles in the *Annals*, academic discussion is encouraged,^2^ and we were pleased to receive four letters from readers regarding this paper.

To afford clarity, brevity and the avoidance of repetition, we asked the authors of these letters to pose specific questions to the study authors. We set out below the questions received from three of the letter authors and the corresponding responses by the original authors.

The Royal College of Surgeons of England has also previously published a response to the paper on its website indicating it does not support surgical care practitioners undertaking laparoscopic cholecystectomies: https://www.rcseng.ac.uk/news-and-events/news/archive/scp-council-discussion-may-2024/

**Questions by K Beatson, University College London, UK**

**Question 1:** Given that the NHS Health Research Authority tool^3^ suggests this work would classify as research, what considerations were given towards ethical approval for the paper? (Could you please elaborate on how this was considered service improvement/evaluation?)

**Response:** This was a retrospective review of the prospectively collected audit data from the logbook of the surgical care practitioner (SCP). The SCP’s training was part of service development and started after she had been in post for nine years. The operations were performed between 2015 and 2019. At the time, the scope of practice was as defined in the 2014 framework.^4^

The surgical care team guidance published by the Royal College of Surgeons of England (RCS England) states: “*All surgical care team members must work within the limits of their competence. The scope of practice of the practitioner, their autonomy and level of supervision needs to be agreed in advance and on a case-by-case basis with the responsible surgeon and ratified by the hospital management. It should reflect their training and experience while allowing for professional development and learning.*”^5^

This was the situation at the time. The last operation was performed in 2019.

The training opportunities were opportunistic, with cases performed as and when the opportunity arose (i.e. suitable case, no trainee).

The NHS Health Research Authority tool states: “*Clinical Audit and Service Evaluation or Service Improvement / Development examine how standard care is delivered or measure improvements in standard care, and by definition all decisions around what treatment regime, care or services to follow and administer are made jointly by the care professional and patient/service user. If this is not the case and treatment regime, care or service is determined by following a set protocol then you should select **YES**.*”^3^

Treatment was not performed under any set protocol and the decisions about treatment were made jointly by the care professionals and patients. The SCP was an experienced clinician who had been appropriately trained and assessed by several experienced surgical trainers as having the required level of knowledge, experience and ability. She had observed and assisted hundreds of these operations prior to this. The operations were supervised by an appropriately experienced doctor in the same way as operations performed by a doctor in training would be. The patients were aware that she was an SCP. There was no independent operating involved.

**Question 2:** Did the authors seek the views of surgeons in training prior to the start of this project, during the project or since its completion?

**Response:** Views of trainees were not specifically sought prior to starting the training. Our department had 12 consultants, 4 registrars and one CT2 doctor. With a 1 in 8 on-call rota, night shifts, annual leave and study leave, there are always operating lists requiring cover. During the period in question, we also had a trainee off for nearly a year as well as academic trainees who only undertook clinical activity for 50% of their time with us. There are often challenges staffing all the lists due to staffing numbers.

The training of the SCP generally did not have an impact on trainees’ access to theatres as there were rarely trainees allocated to those lists. In fact, the presence of the SCP gave flexibility, in that trainees could go to lists that they would most benefit from and the SCP would slot into whichever theatre needed covering for service.

All three consultant authors have been members of the West Midlands/Birmingham and Black Country training committees and regularly attended annual review of competence progression panels where trainees gave feedback on the quality of training in the trust. The SCP post has existed in our trust since 2006 and the deanery has been aware of its existence from the start. There have never been any concerns raised either by surgeons in training or the deanery in the 18 years since the post was established. In light of the concerns raised following publication of our paper, we have sent out a questionnaire to get the opinions of our previous trainees.

**Questions by M Okocha, Royal United Hospitals Bath NHS Foundation Trust, UK**

**Question 1:** What was the necessity for the SCP to be trained in cholecystectomies (i.e. consultant gaps, waiting list pressures, management decision)?

**Response:** The SCP completed her training in 2006. Following this, she spent the subsequent nine years observing, assisting and performing surgery. She spent four days a week in theatre amassing a huge amount of experience. She also demonstrated excellent surgical skills, good judgement and excellent awareness of her limitations. By this point, she had become fully competent at lumps and bumps, umbilical and groin hernias, and had been teaching senior house officers (SHOs) and junior registrars for several years. The reasons for starting the training were partly for further development of the SCP, who had demonstrated the skills, knowledge, attitude and experience to learn the procedure.

Second, it was felt that expanding the skillset of a permanent member of staff would benefit the department by increasing flexibility in covering operating lists and also providing experienced support for more senior trainees as well as teaching and support for those who were more junior.

**Question 2:** Were the training cases opportunistic (i.e. no trainees available, no foundation doctors or core trainees available – although I note there were core trainees holding the laparoscope) or were they scheduled?

**Response:** The cases were performed opportunistically in theatre lists with senior specialty and associate specialist (SAS) doctors or consultants. Seven of the 170 cases were assisted by doctors in core training with a consultant supervising unscrubbed. Staff numbers in our NHS trust mean that staffing all lists can be challenging. We endeavour to place trainees in lists that would benefit them the most. The SCP would cover whichever list she was needed in. The SCP was not given cases in preference to those in training.

**Question 3:** It was noted that these cases were performed between June 2015 and November 2019. Is the SCP still performing independent cholecystectomies now in 2024?

**Response:** The published series includes all the cases performed by the SCP. The last procedure was performed in 2019 and she has not performed any since then.

**Questions by M El Boghdady, St George’s University Hospitals NHS Foundation Trust, UK**

**Question 1:** What is the rationale for upgrading the SCP beyond their framework/scope of practice? Who was regulating their independent practice? What is the need to train an SCP to perform surgeries when there are also surgeons in training and SAS doctors in their department?

**Response:** The SCP role in our trust was part of the pilot scheme introduced by the Department of Health and RCS England. The trust was approached about creating an SCP post by RCS England in 2003. The SCP (who was already an experienced theatre sister) subsequently completed a two-year programme at the University of Greenwich, after which she underwent in-house training spending four days a week in theatre.

As detailed in our paper, the operations reported in this series were performed between 2015 and 2019. The framework in place at the time was the 2014 version.^4^ The 2022 framework^6^ cannot be applied retrospectively. RCS England’s surgical care team guidance states: “*Senior surgical care practitioners can potentially perform surgical procedures independently at a level equivalent to a core surgical trainee year two level (and sometimes up to a specialty trainee year three level).*”^5^ This refers to *independent* practice.

The laparoscopic cholecystectomies described in our paper were not independent practice. They were supervised by consultants and senior SAS doctors. The cases assisted by SHO-grade doctors were supervised by unscrubbed consultants. The practice was regulated by the clinical director as the line manager.

The SCP had demonstrated excellent knowledge, insight, judgement and surgical ability during the 12 years of training and practice leading up to being trained to perform laparoscopic cholecystectomy. Having demonstrated these skills and aptitude for several years, the consultants felt that it would be good development to proceed with the training.

Our department had 12 consultants with 4 registrars, 3 SAS doctors and a teaching fellow as middle-grade doctors. There was one general surgery CT2 doctor on the SHO rota. With a 1 in 8 on-call rota, night shifts, annual leave and study leave, there are regularly gaps in the rota. In addition, during that period, we had a trainee off on maternity leave for most of the placement and academic trainees who were 50% clinical, as well as a period when the deanery was unable to fill all the posts.

**Question 2:** The authors are consultant trainers and one of the authors is known to be a training programme director. How would they justify this practice to Health Education England (HEE)/the Intercollegiate Surgical Curriculum Programme when their trainees do not meet their numbers or competences in laparoscopic cholecystectomy procedures?

**Response:** The cases were not taken from surgeons in training. If a trainee was present, they would always take priority. These were service lists where the SCP was with either the consultant or SAS doctor alone. The presence of the SCP provided flexibility as she could be put into any theatre list, allowing us to place trainees in lists that would benefit their training rather than just providing service.

In the 18 years since the SCP role was introduced, we have not had any concerns raised about operating opportunities or the quality of training in our trust from trainees or the deanery. The deanery has been aware of the role since its introduction in 2006.

**Question 3:** Would it not be expected from consultant trainers and training programme directors to take every opportunity to minimise the impact of rotational training on surgical trainees, rather than using it against the trainees?

**Response:** The training of the SCP was never prioritised over the needs of surgeons in training. The statement in our paper referring to reduced stress relates to the fact that having a familiar assistant makes surgery easier. This is not unique to our department and is highlighted in the 2016 RCS England publication *A Question of Balance: The Extended Surgical Team* (pages 58–60).^7^ Our department has always done everything possible to facilitate and deliver training.

**Question 4:** Did patients fully understand that their operations would be performed by a non-medical practitioner and that performing such surgical procedures is out of their scope of practice, including consenting for surgery?

**Response:** The consent process was undertaken by the SCP wearing an identity badge and nurse’s uniform. She always introduced herself as a nurse. Consenting is within the scope of practice of an SCP. RCS England’s surgical care team guidance states: “*All surgical care team members must work within the limits of their competence. The scope of practice of the practitioner, their autonomy and level of supervision needs to be agreed in advance and on a case-by-case basis with the responsible surgeon and ratified by the hospital management. It should reflect their training and experience while allowing for professional development and learning.*”^5^ There is no mention of any specific procedures in the guidance.

One of the recommendations from the 2016 RCS England publication *A Question of Balance: The Extended Surgical Team* reads:^7^

“*The College and HEE should work with NHS employers to develop guidance, aimed at surgeons and employers, on the following:*

• *Indemnity arrangements (for physician associates in particular)*
• *Governance – including on involving non-medical practitioners in clinical governance mechanisms and team infrastructure*
• *Accountability mechanisms – including with regard to line management arrangements for non-medical practitioners undertaking medical roles*
• *How to define the parameters of scope of practice for non-medical practitioners – including what is meant by ‘independent operating’ and the level of supervision expected*”

The limits of the scope of practice were not clearly defined at the time when our SCP performed these operations.

**Question 5:** Did the authors not find it unfair and humiliating for surgeons in training to be holding the camera or a fundus retractor for a non-medical practitioner who did not go to medical school and did not have any structured training or experience of any of the challenges faced during said training? Did the authors consider reviewing the trainees’ training opportunities and ask about the trainees’ satisfaction in their department?

**Response:** First, the assumption of the lack of structured training is incorrect. As previously mentioned, the trust was asked to develop this role as a pilot for the Department of Health and RCS England. The structure of the programme came from the Department of Health and RCS England, not the trust.

The SCP completed a two-year course at the University of Greenwich studying anatomy, physiology and pathology, among other subjects. She subsequently had formal supervised training with assessment and feedback. She was regularly involved in audit and continuing professional development. She attended the RCS England *Basic Surgical Skills* and specialist registrar skills courses, as well as a laparoscopic skills course. Spending 4 days a week in theatre for 40 weeks works out as over 1,200 theatre hours per annum. This is a vast amount of experience in observing, assisting and performing surgery. The first laparoscopic cholecystectomy in the series was performed 9 years after she qualified and the last one was 14 years after qualification.

One would hope that for a doctor in their first year of surgical training to assist, observe and possibly perform parts of a procedure with such an experienced operator while being observed by a consultant would not be considered humiliating. Our trainee opportunities are regularly reviewed and deanery feedback has always been excellent. As far as we are aware, there have been no concerns raised in the 18 years that the SCP post has been in existence. Many surgeons in training have expressed positive opinions on the SCP role. The department has always had a good reputation for training. One of the authors was a national finalist at the 2022 Association of Surgeons in Training Silver Scalpel awards for outstanding contribution and excellence in surgical training, and was also on the final shortlist for the 2021 Doctors Academy award for outstanding commitment to medical education. Both are national awards nominated by trainees.

**Question 6:** In case of complications, who would be responsible and who would lead the duty of candour?

**Response:** As part of her training, the SCP attended the RCS England *Basic Surgical Skills* and specialist registrar skills courses as well as a laparoscopic skills course. She performed 170 operations under supervision: 163 with the trainer scrubbed and 7 with the trainer unscrubbed. This was after having observed and assisted several hundred of these procedures since qualifying. A total of 110 procedures is the requirement for certification in oesophagogastric and hepatopancreatobiliary surgery. Responsibility and duty of candour would lie with the responsible consultant, as it would if they were supervising a doctor in training.

As previously mentioned, the limits of scope of practice were not clearly defined at the time these procedures were performed. The operations were performed under supervision, not independently. The SCP underwent formal training, as required at the time. The procedures were assessed and she had procedure-based assessments signed off as level 4 by three different consultants. No laparoscopic cholecystectomies have been performed by the SCP since 2019.

We fully accept and adhere to current RCS England guidance.^5,6^
